# Design Principles for PFAS Adsorption in Three-Dimensional Covalent Organic Frameworks

**DOI:** 10.1021/acs.jpcc.6c01608

**Published:** 2026-04-08

**Authors:** Daniel D. Mottern, Andrei L. Kolesnikov, Gennady Y. Gor, Joshua Young

**Affiliations:** † Otto H. York Department of Chemical and Materials Engineering, 5965 New Jersey Institute of Technology, Newark, New Jersey 07102, United States; ‡ Institut für Nichtklassische Chemie e. V., Permoserstraße 15, Leipzig 04318, Germany; § Matlantis Inc., 1 Broadway, Cambridge, Massachusetts 02142, United States

## Abstract

Recent concerns over the accumulation of per- and polyfluoroalkyl substances (PFAS) in surface and groundwater sources have stimulated research into novel porous materials for selective PFAS adsorption. Covalent organic frameworks (COFs) represent one of the promising families of materials for this application. Here, we investigated the effects of the chemistry and structure of three-dimensional COFs on the adsorption of perfluorooctanoic acid (PFOA), a commonly seen PFAS molecule. Through Monte Carlo (MC) simulations, we found that nitrogen-based COFs tend to show high potential for PFAS adsorption. We also see that the porosity of the COF pores has a significant effect on PFOA adsorption, with higher porosity structures exhibiting lower potential for PFAS adsorption than those with moderate porosity. We additionally investigated the effects of COF functionalization with −CF_3_ and −NH_2_ functional groups, showing that both functional groups strengthen interactions between the PFOA molecule and COF, but may decrease the porosity needed for effective adsorption of PFOA. For COFs with large enough pores, the addition of these functional groups can greatly improve the adsorption of PFOA and could allow for the improved capture of PFAS from aqueous environments.

## Graphical abstract

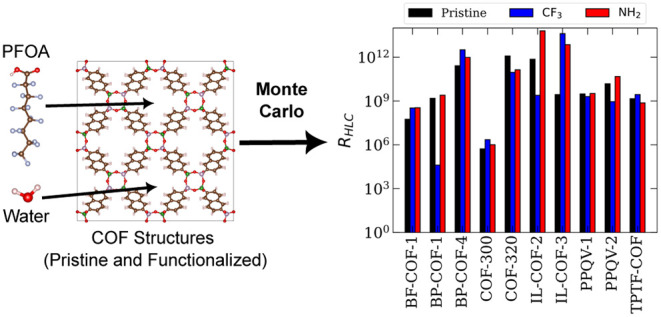

## Introduction

Per- and polyfluoroalkyl substances (PFAS) are a large family of chemicals which are notable for their fluorinated carbon backbone, lending high thermal and chemical stability.^1^ These properties have led to their use in various industries and applications, including wastewater treatment, fire fighting foams, nonstick cooking surfaces, and textiles.^1–3^ While this high stability allows PFAS molecules to serve as surfactants, it also allows the molecules to resist degradation and decomposition in nature.^1^ This has led to these so-called “forever chemicals” accumulating in freshwater and groundwater sources, as well as within soil.^4–6^ From there, PFAS can enter the human body through drinking water and bottled beverages.^7,8^

Human and animal studies on the toxicity of PFAS have shown connections to various adverse health effects, including increased risks of kidney, testicular, and breast cancers^9,10^ neurological disorders^11^ and higher incidence of childhood diabetes and adiposity.^12,13^ Concerns regarding PFAS accumulation in the environment, as well as these potential health hazards, have spurred interest in new materials and methods for the removal of PFAS from water. Popular methods for remediating PFAS from aqueous environments include degradation and adsorption.^14,15^

Particular attention has gone toward adsorptive methods for PFAS removal due to the low cost and high scalability of the materials being considered.^14^ One of the most readily available materials, activated carbon, is an incredibly inexpensive material that has been used for water remediation prior to the newer concerns of PFAS.^14^ Despite its wide use, activated carbon has very slow adsorption kinetics for long chain PFAS, and has been shown ineffective at adsorbing short chain PFAS altogether.^16^ Other carbonaceous adsorbents, such as carbon nanotubes and graphene sheets, improve on the performance of activated carbon, due to their higher surface areas and porosity, but are significantly more expensive.^17^ Polymeric materials, such as ion exchange resins and polymer networks, have additionally shown potential as adsorptive materials, with some showing stronger PFAS capture performance than activated carbon, but the regeneration has been a hindrance to progress with these materials.^18^

One novel group of materials that have shown promise are porous crystalline materials, including zeolites, metal organic frameworks (MOFs), and covalent organic frameworks (COFs).^19,20^ These families of materials have attracted attention due to their high internal surface areas and pore volumes, as well as their capabilities for further functionalization to improve their properties.^20^ Several MOFs, such as NU-1000, MIL-101­(Cr), and UiO-66, have been experimentally studied for their PFAS removal capabilities,^21–23^ while zeolite Beta has been researched extensively, showing faster and larger PFAS uptake compared to activated carbon.^19^ COFs, meanwhile, have received some attention, but it has been limited to a small number of structures.^24,25^

More recently, computational molecular modeling has been used to investigate the adsorption capability of COFs without the need to synthesize the structures. One example comes from Zhang et al., which utilized machine learning-assisted molecular simulations to study the adsorption energy of perfluorobutanoic acid (PFBA), a short-chain PFAS molecule, in a number of hypothetical COF structures.^26^ COFs were screened based on several structural parameters, such as the pore-limiting diameter and porosity. Monte Carlo simulations were used to build the training data set for a machine learning model, which was then able to predict the adsorption performance of 65,000 hypothetical COF structures.^26^

Beyond the pristine structures, functionalization has shown potential to improve the adsorptive removal of PFAS in both MOFs^27^ and COFs.^25^ Two functional groups in particular, trifluoromethyl groups (−CF_3_) and amine groups (−NH_2_), have been studied experimentally for their effects on the strength and nature of PFAS adsorption.^25,28,29^ Trifluoromethyl groups were found to improve adsorption by introducing fluorine–fluorine interactions between the adsorbate and the PFAS tail group.^28,29^ Meanwhile, amine functional groups target the often anionic headgroup of the PFAS molecules.^25,29^ However, these functionalization studies often focused on specialized COFs that were built with their functional groups in mind rather than altering existing COF structures. The question remains how these functional groups can be applied to more well-known and commonly synthesized COFs.

In this work, we investigate 96 three-dimensional COF structures using Monte Carlo (MC) simulations to explore their potential to remove PFAS from water. We study perfluorooctanoic acid (PFOA), one of the most commonly seen PFAS molecules in freshwater and groundwater, to analyze how this molecule interacts with the COF structures. Henry’s law coefficients were calculated for both PFOA and water in each COF, and these values are used to compare and assess the performance of different COFs. Further, 10 COF structures were chosen for functionalization with trifluoromethyl and amine groups. We find that these functional groups have the potential to improve PFAS adsorption performance, but that it is dependent on the pore size and chemistry of the initial COF.

## Computational Methods

In this study, we considered the nearly 900 experimentally synthesized COF structures collected in the Curated COF Database, which have been optimized using DFT.^30^ We chose to focus on the available three-dimensional COF structures in this database, due to their generally higher internal surface areas and water stability compared to layered two-dimensional structures.^31,32^ This makes them more advantageous for the adsorption of aqueous PFAS.^31^ This left us with 120 three-dimensional COFs, which included more common boron- and nitrogen-based COFs^33^ as well as more novel structures, such as borophosphonate COFs^34^ and COFs that feature trace amounts of metallic elements.^35^ Of these 120 COF structures, seven were discarded due to the lack of geometrically optimized structures in the database^30^ and another 17 received Monte Carlo simulations as described below, but due to their low pore diameters resulted in negligible adsorption, and were removed from further testing. The remaining 96 COF structures were studied using Monte Carlo simulations, with a small selection of structures chosen as candidates for functionalization with trifluoromethyl (−CF_3_) and amine (−NH_2_) functional groups. The workflow for this study is shown in Figure 1.

**Figure 1:**
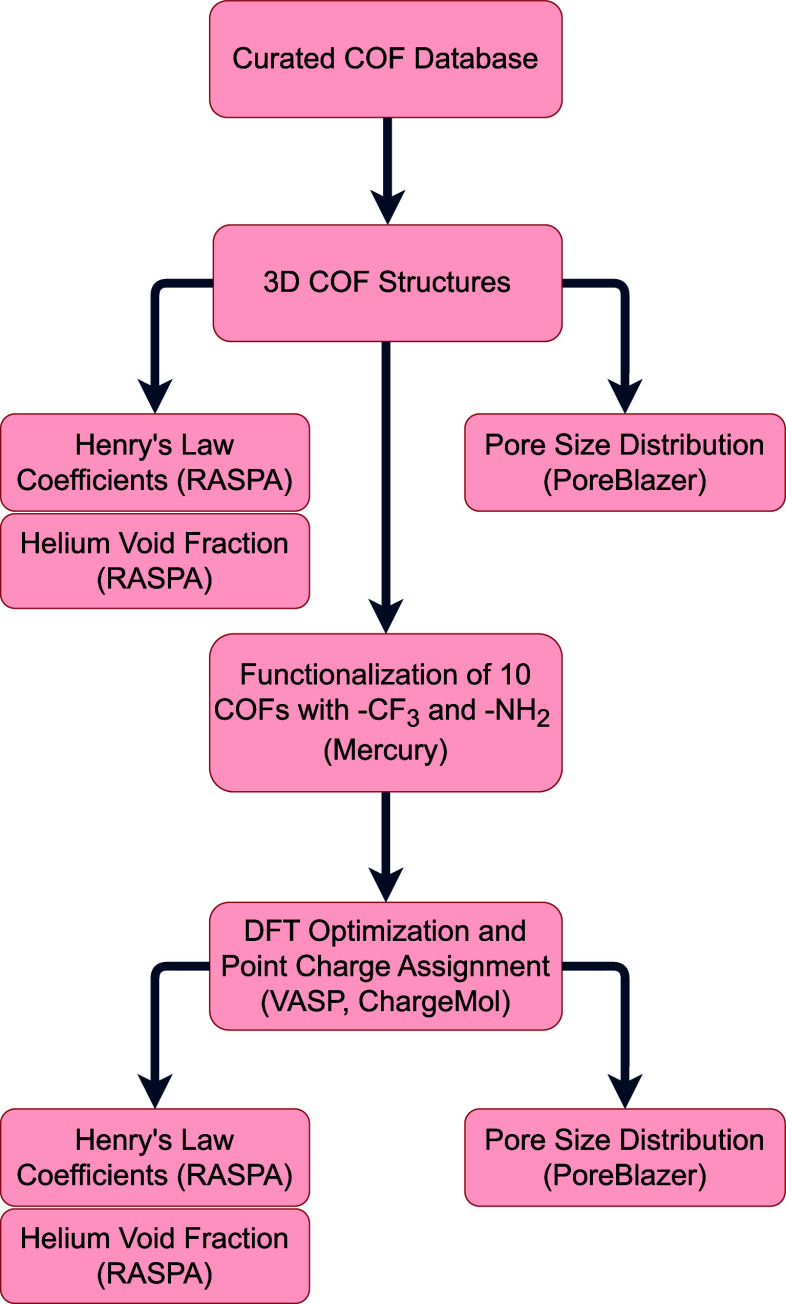
Computational workflow followed in this paper. For relevant steps, corresponding computational tools are mentioned. This includes; the Curated COF Database;^30^ RASPA^36^ a classical Monte Carlo package; Poreblazer^37^ a pore size analysis software; Mercury^38^ a visualization software; VASP^39–42^ a Density Functional Theory package; and ChargeMol,^43–45^ a DDEC charge analysis code.

### Force Field Parameters

The force fields used in the simulations in this paper utilize Lennard-Jones (LJ) potentials to compute the short-range atomic interactions, and Coulomb potentials for the long-range electrostatic interactions of the system. These were calculated with the equation:

1 $$U\left(r_{ij}\right)=4\epsilon_{ij}\left[{\left(\frac{\sigma_{ij}}{r_{ij}}\right)}^{12}-{\left(\frac{\sigma_{ij}}{r_{ij}}\right)}^{6}\right]+\frac{q_{i}q_{j}}{4\pi \epsilon_{0}r_{ij}}$$

where *U* is the total energy between interacting atoms *i* and *j*, at a distance of *r*_*ij*_ from one another. The first term of the equation defines the contribution of the LJ potential to the total energy, where ϵ_*ij*_ and σ_*ij*_ are the well depth of the LJ potential energy curve, and the interatomic distance at which the interatomic potential energy between atoms *i* and *j* is zero, respectively. The second term describes the Coulombic interactions of the system, with *q*_*i*_ and *q*_*j*_ being the partial charges of atoms *i* and *j*, and ϵ_0_ being the vacuum permittivity.

The LJ parameters for atoms in the COF structures were taken from the UFF force field^46^ while LJ parameters acting between different atoms in the systems were calculated using the Lorentz–Berthelot mixing rules. The UFF model was chosen over more system-specific, DFT-derived force fields due to its wide applicability, which can account for the vast structural and chemical variety of the 3D COFs being studied. The UFF model shows reasonable agreement with experimental gas adsorption data on COFs^47^ and has been widely used in previous computational studies.^48–51^ For the COF structures available in the Curated COF Database, the optimized structures and partial charges were taken from the database.

The COFs were treated as rigid structures for all Monte Carlo simulations. While all nanoporous materials show some degree of flexibility during the adsorption process, resulting in adsorption-induced deformation, most materials show strain on the order of fractions of a percent, and these strains are neglected.^52^ Due to their covalent bonds, most COFs, unlike MOFs, have quite rigid structures, not showing appreciable flexibility, with only a few exceptions reported in the literature.^53–55^ To our knowledge, there are no molecular simulation studies which take into account the flexibility of COFs during adsorption, and so we are also considering the structures rigid.

For the adsorbate molecules, PFOA and water, two different models were used. For water, the TIP4P-Ew model was utilized.^56^ This model was selected based on its high degree of consistency with experimental results compared to other water models, such as SPC or OPC models.^57^ The bond lengths and angles for water molecules were also fixed. We chose the PFOA model developed by Erkal et al.^58^ which treats the −CF_2_ and −CF_3_ groups in the fluorinated carbon tail as united atoms, but explicitly defines the atoms for the carboxyl headgroup. The chemical structure and united atom model of PFOA can be seen in Figure 2. The bond lengths for the PFOA molecule were constant, but harmonic bending and dihedral torsion were modeled for the bonds. Tail corrections were not used. The parameters were derived from available force fields for molecules with similar structures, such as perfluoroalkanes and carboxylic acid.^59–61^

**Figure 2:**
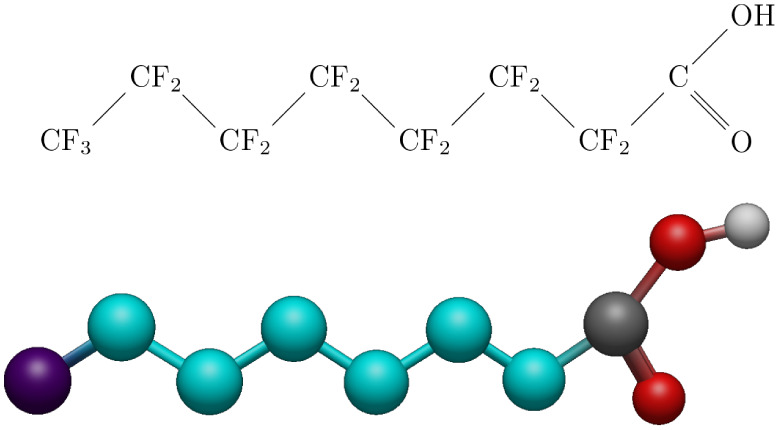
(Top) Chemical formula of PFOA. (Bottom) United atom model of PFOA, with purple representing CF_3_, cyan as CF_2_, gray as C, red as O, and white as H.

A set of sample files for simulations conducted in this study, including the force field parameters for PFOA, are available in the Supporting Information. We validated the force field using a molecular dynamics simulation in the NPT ensemble on pure PFOA. The simulation ran for 10^4^ time steps of 0.001 ps, and the density was calculated at a temperature of 298 K and pressure of 1 atm. Ewald summation was employed to capture long-range interactions, with a precision of 10^–6^. Additionally, we calculated Henry’s law coefficients for MOFs studied by Erkal et al. in their initial paper to compare our values.^58^

Although PFOA and other acidic PFAS molecules are expected to dissociate from their acidic hydrogen in the presence of liquid water, the above force field describes PFOA in its protonated form. This is due to the presumed hydrophobic nature of the pore surfaces of the COF structures, which we intend to show through the calculated water Henry’s law coefficient (HLC) values. In hydrophobic pores, this surface hydrophobicity is expected to result in negligible amounts of water making their way into the pore structure.^58^ In this case, PFOA would then remain in its neutral, protonated form. This assumption however would be dependent on the size of the pores, as larger pores would see more affinity for water entering the pores than smaller pores. In the case of sufficiently large pore sizes, the development of force field parameters for the anionic form of PFOA may be necessary for comparison.

### Henry’s Law Coefficient Simulations

Monte Carlo (MC) simulations were performed using the RASPA molecular simulation software package version 2.0.39.^36^ All simulations employed a 12 Å cutoff distance for LJ and Coulombic potentials. The unit cells were simulated such that all sides of the unit cell were longer than twice the cutoff distance. MC simulations were utilized in order to calculate the HLCs of both PFOA and water inside the COF structures. This was done by following the Widom insertion method^62^ which randomly samples the properties of a singular molecule being inserted at a random position within the pore. Unlike traditional MC simulation methods, the molecule is not “permanently inserted” in the Widom insertion method, but properties such as the energy, chemical potential, and Henry’s law coefficient can be calculated. By sampling 10^5^ insertion positions, we can find the HLC of an adsorbate molecule inside the COF pore. For each randomly sampled position, the HLCs for PFOA and water are calculated by RASPA following the equation,

2 $$K_{H}=\frac{1}{RT\rho_{f}}\frac{\left\langle W\right\rangle }{\left\langle W^{\mathrm{ig}}\right\rangle }$$

where ρ_f_ is the density of the COF framework, ⟨*W*⟩ is the Rosenbluth weight of the molecule in the COF pore, and ⟨*W* ^ig^⟩ is the Rosenbluth weight of the molecule in the ideal gas phase, which serves as a reference point.^63^ These values for each molecule are averaged over the sampled positions. Next, the ratio of the Henry’s law coefficients of PFOA and water were taken, and used to select a small group of COF structures to functionalize, listed in Table 1.

**Table 1:** Names and Chemical Formulas for Candidates for Functionalization with −CF_3_ and −NH_2_

COF Name	Chemical Formula	Refs
BF-COF-1	C_78_H_60_	^67^
BP-COF-1	C_48_H_32_B_8_P_8_O_24_	^34^
BP-COF-4	C_80_H_48_B_8_P_8_O_24_	^34^
COF-300	C_164_H_112_N_16_	^53^
COF-320	C_53_H_36_N_4_	^68^
IL-COF-2	C_212_H_144_N_16_	^69^
IL-COF-3	C_260_H_176_N_16_	^69^
PPQV-1	C_180_H_108_N_12_	^70^
PPQV-2	C_216_H_132_N_12_	^70^
TPTF-COF	C_74_H_44_N_4_	^71^

### Functionalization of COF Structures

Of the 96 COF structures which yielded Henry’s law coefficient results, 10 structures, given in Table 1, were chosen for functionalization with trifluoromethyl and amine groups. COF-300, COF-320, and BF-COF-1 were chosen based on existing experimental studies showing their capability for removing aqueous pollutants.^64–67^ BP-COF-1 and BP-COF-4 were selected as the highest performing non-nitrogen-containing COF structures. IL-COF-2 and IL-COF-3, PPQV-1, PPQV-2, and TPTF-COF were picked as five structures in the CHN family of COFs which showed promise for PFOA adsorption. Functional groups were manually added using the Mercury software system^38^ to replace terminal hydrogen atoms on the COF linkers with functional groups, either −CF_3_ or −NH_2_. In order to both limit the effects of these functional groups on the available pore diameters, and to retain a system size that can be reasonably optimized using DFT, only 10–15% of linker hydrogen atoms were functionalized. Experimental studies have shown that excess functionalization can begin to hinder the uptake of PFAS molecules.^25^ The 10–15% functionalization was chosen as a reasonable range of functionalization to be able to investigate the effects of the groups while avoiding the pore blockage arising from too high a concentration. This translates to between one and three functional groups per linker, depending on the COF pore size. The functional groups were placed within the COF pore such that they are oriented toward the center of the pore, making them more accessible to PFOA and water molecules. However, the exact pattern of functionalization, including the total number of functional groups per COF pore and their arrangement, will vary significantly, especially in experimental synthesis. The variations and randomness of a realistic functionalized COF will have an effect on the actual adsorption strength, but are difficult to recreate in simulations that utilize periodic boundary conditions. Thus, the functionalized structures analyzed in this study represent one possible arrangement for these functional groups, but allow for an understanding of their effect on PFAS adsorption.

After introducing functional groups to the COF, we employed geometric and electronic optimization using periodic density functional theory (DFT), which was carried out with the Vienna ab initio Simulation Package version 6.4.2 (VASP 6.4.2).^39–42^ The PBE functional was utilized^72^ with a cutoff energy of 800 eV. A force convergence of 0.001 eVÅ^–1^ was selected, as well as a 1 × 1 × 1 Γ-centered k-mesh. After geometrically optimizing the functionalized COF structures, the partial atomic charges were calculated using the ChargeMol software package, which follows the Density Derived Electrostatic and Chemical (DDEC6) method.^43–45^ This process is consistent with the method described in the database^30^ to optimize and assign point charges to the pristine COF structures in this study. The functionalized and geometrically optimized COFs then underwent the same process as their pristine counterparts to determine the HLC ratio between PFOA and water.

### Pore Size Analysis

In order to further understand the relationship between the pore geometry of a given COF and its ability to adsorb PFOA, the pore size distribution (PSD) of the 10 selected COFs before and after functionalization were computed. These PSDs were created using the PoreBlazer v4.0 software, which utilizes a nitrogen probe to explore the interior surface of the COF.^37^ The PoreBlazer software was benchmarked against both RASPA and another common structural analysis package, Zeo++, and was found to achieve results consistent with both for a variety of properties.^37^ In addition, we calculated the porosities for a handful of pristine COF structures that were the focus of this work using RASPA, PoreBlazer, and Zeo++ to form a comparison of our own. The resulting porosities are shown in Table S1. The three methods produce results that are consistently within 5% of each other. The default nitrogen probe settings at 298 K were selected for these pore size distribution calculations. 500 samples were taken per framework atom, with a maximum histogram bin size of 0.25 Å. These PSDs allow us to see how the introduction of the −CF_3_ or −NH_2_ functional groups affect the diameters of the COF pores, and how these shifts might relate to the COF’s ability to adsorb PFOA.

## Results

Prior to beginning any PFOA-COF simulations for this study, we first performed a molecular dynamics simulation in the NPT ensemble on pure PFOA at 298 K and 1 atm, showing that the force field from Erkal et al. replicated the amorphous solid density of PFOA well (1830 kg m^–3^ in our calculation vs 1800 kg m^–3^ experimentally).^58^ Additionally, we reproduced the Henry’s law coefficients for PFOA with selected MOFs from Erkal et al.^58^

After verifying the force field parameters, we began working with the 96 three-dimensional COF structures available on the Curated COF Database.^30^ These structures represented a variety of pore diameters and chemical compositions. Following the study by Erkal et al.^58^ our primary criterion for a suitable COF adsorbent was a high ratio of the Henry’s law coefficients of PFOA to water,

3 $$R_{\mathrm{HLC}}=\frac{H_{\mathrm{PFOA}}}{H_{H_{2}O}}$$

with *H*_PFOA_ and $H_{H_{2}O}$ representing the Henry’s law coefficients of PFOA and water respectively. Based on the low concentrations of PFOA in water sources^17^ the solubility of PFOA in the COF pore can be modeled following Henry’s law, making *R*_HLC_ a way of comparing the relative solubility of PFOA and water in the COF pore. A higher *R*_HLC_ thus would mean a high Henry’s law coefficient for PFOA, indicating high removal capacity, and a low Henry’s law coefficient for water, suggesting a hydrophobic pore interior.^58^ We first looked for trends in *R*_HLC_ for the COF structures, which are summarized in Figure 3. From these results, we selected 10 COF structures that showed a capacity for adsorbing PFOA to functionalize with −CF_3_ and −NH_2_ functional groups. These structures were optimized, and the *R*_HLC_ values were calculated. These values are shown in Figure 4, alongside their pristine *R*_HLC_. Finally, the porosities and pore size distributions for the pristine and functionalized COFs were analyzed to gain further insight on how functionalization affects the adsorption properties of these COFs.

**Figure 3:**
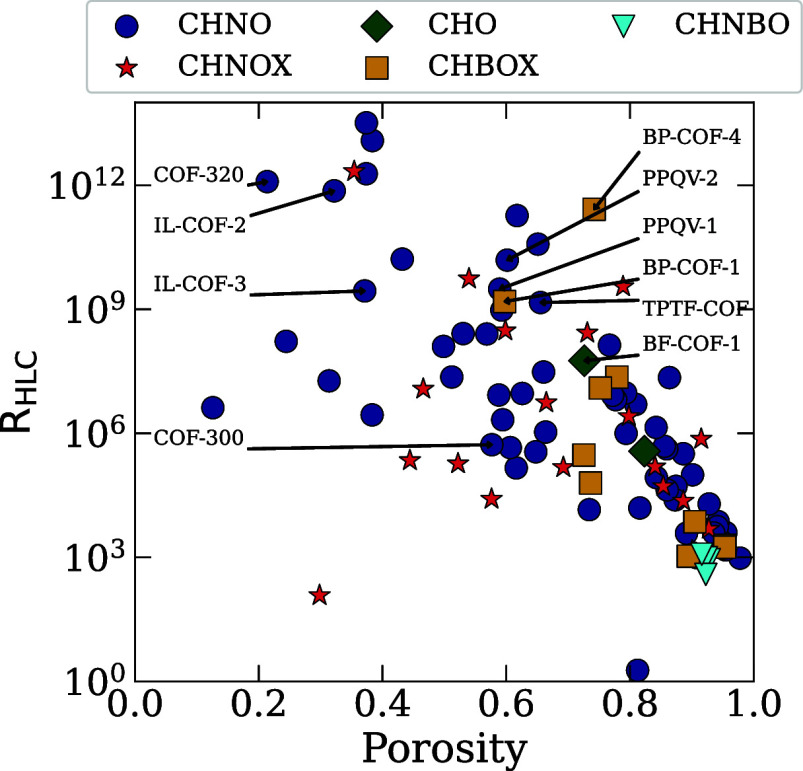
Scatterplot of *R*_HLC_ against porosity for five families of COF structures. The CHNO COF family is marked in dark blue, CHNOX in red, CHO in dark green, CHBOX in yellow, and CHNBO in cyan. COFs chosen for functionalization with trifluoromethyl and amine groups have been labeled.

**Figure 4:**
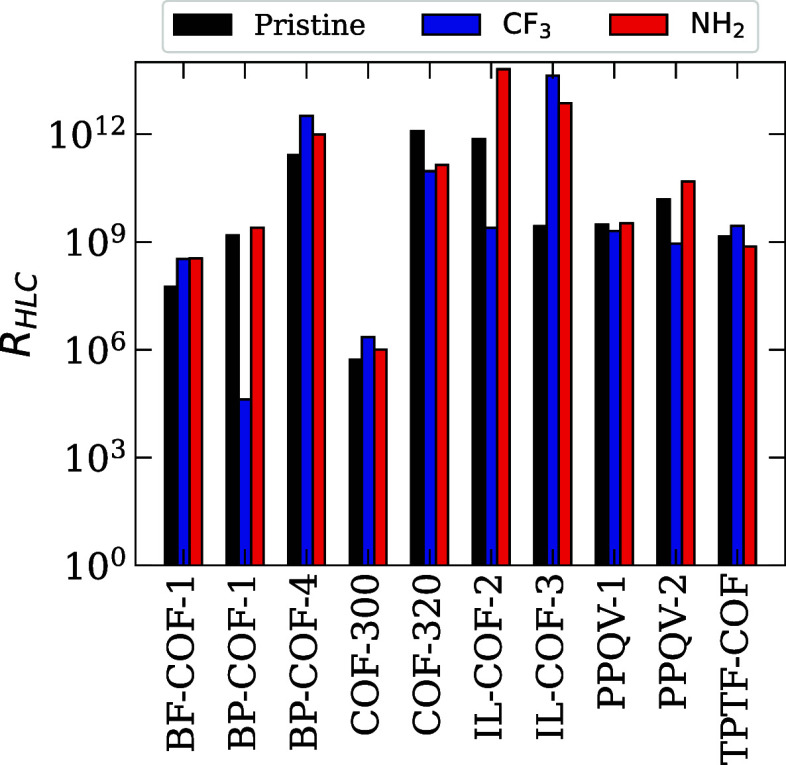
*R*_HLC_ values for pristine, CF_3_-functionalized, and NH_2_-functionalized versions of the ten selected COFs.

### Pristine COF Henry’s Law Coefficients

We computed the Henry’s law coefficient of both water and PFOA in the remaining COF structures, as well as calculated the *R*_HLC_ values. These values are tabulated for all 96 COF structures, and are provided in Supporting Information. Of the 113 optimized COF structures available in the database^30^ not all were suitable for the adsorption of PFOA. Since the Widom insertion method samples random adsorption positions without “truly placing” the molecule in the COF pore, large molecules and small pore sizes can lead to results that approach extreme highs and lows. In the case of the Henry’s law coefficient of PFOA, we found that 17 COF structures with very low pore diameters produced HLC values that approached zero, indicating that the molecule would not realistically fit into the COF pore. Due to the size restriction, these COF structures were removed from future consideration, leaving us with 96 total COFs to investigate.

We next analyze the trends in the *R*_HLC_ values for the COFs through the lens of two important factors for the adsorption of PFOA in a COF structure. The first is the porosity of the COF, described by the equation

4 $$\phi =\frac{V_{\mathrm{void}}}{V_{\mathrm{total}}}$$

ranging from 0 for a nonporous material to 1 for a hypothetical pure void with no matter. Since this is not realistic, a COF structure will never achieve a porosity of 1. However, for COFs with very high pore volumes and simplistic ligands, such as JUC-556-HZxE, porosities as high as 0.97 can be achieved. The second factor is the chemical composition of the COFs, which will affect the adsorption strength of a molecule within the COF pore. By combining these two, we can look at the trends in how the *R*_HLC_ values vary with the COF porosity. Further, the COFs have been categorized into five families based on the chemical formulas of the COF (CHO, CHNO, CHNOX, CHBOX, and CHNBO). CHO, CHNO, and CHNBO COFs contain only the elements contained in their respective family’s name—carbon, hydrogen, oxygen, nitrogen, and boron. CHNOX and CHBOX feature their respective elements, but with an additional element, X; the majority of COFs in these two categories incorporate elements such as X = fluorine, chlorine, phosphorus, and sulfur, but a handful also include metals and metalloids, such as nickel, copper, and silicon.

Figure 3 shows a scatterplot of *R*_HLC_ against porosity for the five different COF families. In terms of overall trends for the 96 COFs, there is a decrease in the *R*_HLC_ values as the porosity increases. This trend is especially pronounced at very high porosity, between 0.7 and 1.0, where many of the COFs are clustered together. For COFs with porosity below 0.7, the majority still have high *R*_HLC_ values, but we see much higher variation in the exact magnitude, and the COFs are not as tightly clustered. In this low range of porosity, we see some of the highest performing COF structures, such as 3D-COF-10, COF-320 and IL-COF-2, as well as COFs with middling strength. This is due to the hydrophobic nature of the interior surfaces of the COF pore. When the surface is hydrophobic, the pore volume near the surface will serve as a depletion region, where water molecules are much less likely to accumulate. In a COF with a smaller pore diameter or porosity, this depletion region will account for a larger portion of the pore volume, while COFs with a larger pore diameter or porosity will have larger volumes that fall outside of the depletion region. In the case of COFs with a higher porosity, we tend to see an increase in the HLC of water, which indicates that there are more available configurations for water inside of the COF pore volume, supporting this trend.

Moving on to trends within COF families, we first have to look at the distribution of the COFs within these groups. The CHO and CHNBO families only have 2 and 4 structures in them each, while CHNO, CHNOX, and CHBOX have 61, 19, and 10 each. As a result, the CHO and CHNBO families do not show any significant trends. However, we do see that both of these families exhibit relatively low *R*_HLC_ values, with the highest being BF-COF-1, a CHO COF with a *R*_HLC_ value of 5.6 × 10^7^.

Looking at the larger COF families, the CHNO and CHBOX COFs seem to follow a similar trend to the overall plot, with higher porosity COFs showing generally lower *R*_HLC_ values. For the CHNO COFs, this trend takes place over a wide ranges of porosities. On the other hand, we see that the CHBOX family COFs only cover a range of porosities between 0.6 and 1.0 while still agreeing with the overall trend. Meanwhile, the CHNOX family of COFs does not align very well with the overall trend. These COFs have porosities ranging between 0.3 and 1.0, and while the lower porosity COFs have the highest performing COF from this category, in TPB-COF-F, it also shows one of the weakest COFs of any category in CCOF-15. Meanwhile, the remaining COFs all show relatively middling strength, regardless of their porosity.

Of the six COFs with the highest *R*_HLC_ values, all six had porosities below 0.4. Five structures are members of the CHNO COF family, while the one remaining COF is in the CHNOX family. Extending to more of the high performing structures, we can see that 17 total COFs exhibited *R*_HLC_ above 10^9^, with 12 falling into the CHNO family, 3 in CHNOX, and 2 in CHBOX. This can likely be attributed to the more hydrophobic nature of nitrogen-based COFs compared to boron-based COFs.^33^ The two CHBOX structures that performed well are both from a family of boron–phosphorus COFs, which increase the valency of the COF, and result in stronger adsorption.^34^

With this, we have established that the chemistry, pore size, and porosity will have an effect on the hydrophobicity of the COF pores, and by extension, on the adsorption of PFOA and water molecules within the pore. The nitrogen-based COFs tend to be more stable in water, and show more hydrophobic behavior, while boron and boron–nitrogen COFs tend to show overall weaker performance, which is consistent with experimental results.^73^ Meanwhile, we see that the *R*_HLC_ of COFs tends to increase at lower porosities, where larger portions of the pore volume exhibit hydrophobic behavior.

### Henry’s Law Coefficients for Functionalized COFs

Now that we have analyzed the trends for pristine COFs, we can investigate how we can modify these structures to improve their performance. To do this, we selected ten COF structures which showed some capability to adsorb PFOA as the bases for functionalization with trifluoromethyl (−CF_3_) and amine (−NH_2_) functional groups. The COFs that were chosen are COF-300, COF-320, BF-COF-1, BP-COF-1, BP-COF-4, IL-COF-2, IL-COF-3, PPQV-1, PPQV-2, and TPTF-COF. Each COF was functionalized by replacing 10–15% of hydrogen atoms on the COF linkers with the chosen functional group, optimized using DFT, and assigned partial atomic charges using the DDEC6 method. The partial charges can give us insight into the changes in the electrostatic potential of our system before and after functionalization. In the CF_3_-functionalized systems, we see that the atoms of the COF backbone tend to become slightly more positively charged compared to the pristine case, while the atoms in the functional group tend to be negatively charged, indicating that the functional groups are acting as electron withdrawing groups. Meanwhile, in the NH_2_-functionalized structures, we see that the nitrogen atoms in both the COF and functionals become slightly more negatively charged, while other elements, such as carbon, become more positively charged. Changes in the electrostatic interactions in COFs have been shown to positively impact the binding energy of both neutral and anionic PFAS molecules.^74^

Once the structures were optimized, we computed the *R*_HLC_ inside the functionalized COF, following the same steps as before, and compared the *R*_HLC_ of the two functionalized structures to that of the pristine COF. The *R*_HLC_ values for the pristine, CF_3_-functionalized, and NH_2_-functionalized COFs are plotted in Figure 4, while the HLCs for PFOA and water are displayed in Figure 5. Looking at the trends in Figure 4, we can see that each COF responds differently to being functionalized. Of the ten COFs, 8 NH_2_-functionalized structures showed a similar or improved *R*_HLC_ value compared to the pristine version, while the remaining two COFs, TPTF-COF and COF-320, showed a slight decrease. As shown in Figure 5, the COFs that showed improvement generally see an increase in the HLC of PFOA compared to the pristine COF, while the HLC of water tends to stay approximately the same, leading to an overall higher *R*_HLC_. This rise in HLC for PFOA can be attributed to improved electrostatic interactions between the PFOA headgroup and the newly functionalized surfaces. For TPTF-COF and COF-320, we see a significant decrease in the HLC of PFOA when functionalized with NH_2_, resulting in increased *R*_HLC_ values.

**Figure 5:**
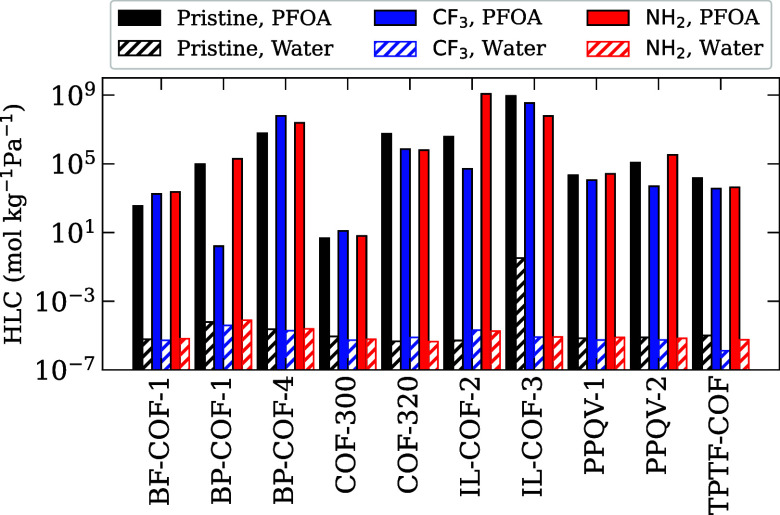
Henry’s law coefficient values for PFOA and water in pristine and functionalized COF structures.

The CF_3_-functionalized structures meanwhile show much more variety. The CF_3_-functionalized COFs outperform their pristine counterparts in five cases (BF-COF-1, BP-COF-4, COF-300, IL-COF-3, and TPTF-COF), with small to severe decreases in *R*_HLC_ for the other five COFs (BP-COF-1, COF-320, IL-COF-2, PPQV-1, and PPQV-2). For COFs that saw improvement, we find moderate increases in the HLC for PFOA, and little change in the HLC for water, as shown in Figure 5. These can be attributed to the −CF_3_ functional groups providing a platform for fluorophilic interactions with the PFOA molecule, as well as making the pore interior more hydrophobic. For the structures that show worse performance postfunctionalization, the most significant change comes from these structures having decreased HLCs for PFOA, with the same or slightly lower HLCs for water.

We can see from these results that the chemistry of the −CF_3_ and −NH_2_ functional groups have the potential to improve the adsorption strength of COFs. However, we cannot predict just from the chemistry of the functional group and COF structure whether or not PFOA adsorption will improve. To get a better idea of how functionalization effects COF performance, we need to look at the porosity and pore diameters of the COF structures.

### Porosity and Pore Size Analysis

In order to provide an additional comparison between the pristine and functionalized COFs, the porosities of each structure before and after functionalization were calculated using RASPA, and the values are given in Table 2. The majority of COFs see a decrease in porosity after functionalization. This is a reasonable outcome, as the functional groups will occupy some of the pore volume that might previously have been accessible for adsorption. However, this is not the case for the COF-300, COF-320, and BF-COF-1 structures. For the COF-300 and COF-320 structures, we see a slight increase in porosity for −CF_3_ and −NH_2_ functionalized structures. For the case of BF-COF-1, we see that the porosity decreases when functionalized with −CF_3_, but increases slightly when functionalized with −NH_2_. These changes in porosity may be a result of how the COF structures rearranged during the DFT geometric relaxations. In these cases, the pores may have further opened during relaxation, leading to slight increases in the porosity.

**Table 2:** Porosities of Pristine and CF_3_- and NH_2_-Functionalized COF Structures

COF Name	Pristine	CF_3_–COF	NH_2_–COF
COF300	0.58	0.64	0.68
COF320	0.21	0.24	0.24
BFCOF1	0.73	0.66	0.77
BPCOF-1	0.60	0.31	0.50
BPCOF-4	0.74	0.62	0.72
ILCOF-2	0.32	0.24	0.27
ILCOF-3	0.37	0.30	0.32
PPQV1	0.59	0.49	0.57
PPQV2	0.60	0.55	0.57
TPTF-COF	0.63	0.25	0.41

The magnitude of the changes in porosity are also largely consistent, with most COFs showing an absolute difference of less than ±10% from the pristine structure. However, the porosity of TPTF-COF decreases from 63% to 41% for the NH_2_-functionalized structure, and to 25% for the CF_3_-functionalized structure. Looking ahead to the pore size distributions in Figure 6 again, we can see that TPTF-COF also shows some of the largest differences in pore radii within the distribution, decreasing from 11 to 12 Å for the pristine case, to 8.5 Å for NH_2_-functionalized, and 7–7.5 Å for the CF_3_-functionalized structure. This large difference in pore sizes may contribute to the large magnitude of difference in porosity for TPTF-COF.

**Figure 6:**
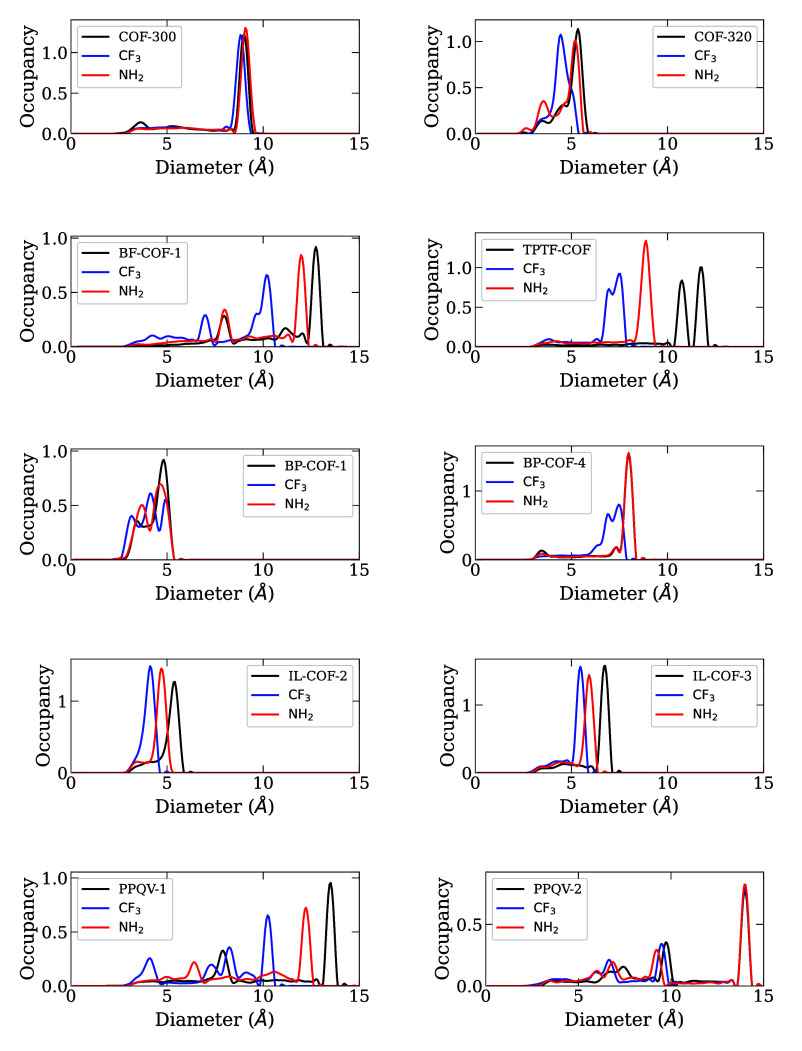
Pore size distributions for pristine and functionalized COF structures.

Our data did not show any clear trend between the porosity changes and the *R*_HLC_ values for PFOA and water in the pristine and functionalized COFs. Even for structures that exhibit similar changes in porosity, the *R*_HLC_ values can vary significantly. This is because the porosity only tells us the relative volume of a pore available for adsorption, and does not provide information about the size of the pores themselves. A 4 Å pore and a 10 Å pore with the same porosity will likely not be able to adsorb the same molecules with the same strength. Because of this, it is important for us to directly investigate the pore sizes of the pristine and functionalized COFs. The pore size distributions of the structures were calculated using the PoreBlazer 4.0 software.^37^ The resulting PSDs from these simulations are shown in Figure 6 below.

There are three trends that we can see in the PSDs for the COFs. For COF-320, BF-COF-1, PPQV-1, IL-COF-2, IL-COF-3, and TPTF-COF, the functionalized COFs all exhibit a shift in PSD peaks toward smaller diameters. This shows that overall, the pore sizes decrease with functionalization, which can lead to PFOA molecules not being able to adsorb in the pores. In COF-300 and PPQV-2, we find that the pore diameters do not change significantly in the functionalized structures. Lastly, for BP-COF-1 and BP-COF-4 the behavior is mixed. In BP-COF-4, the NH_2_-functionalized structure has a nearly identical pore size distribution to the pristine structure, while the CF_3_-functionalized structure shifts toward smaller pore sizes, and there is a change from one large peak to two smaller peaks, indicating more variety in pore diameters. For BP-COF-1, we see some movement toward smaller pore sizes for both functional groups, as well as a difference in peak numbers. The pristine COF features one prominent peak at approximately 4.75 Å and a much smaller peak at 3.25 Å. The NH_2_-functionalized structure has two prominent peaks, at 3.5 Å and 4.5 Å. Finally, the CF_3_-functionalized structure exhibits three peaks, at 3, 4, and 5 Å.

We can use these PSDs to understand some of the *R*_HLC_ results for the functionalized COFs. COF-300, BF-COF-1, BP-COF-4, and IL-COF-3 showed improvement for both −CF_3_ and −NH_2_ functionalization. The pore sizes for these COFs fall between 5.5 Å and 12 Å. Previous studies with functionalized MOFs found that pores in the range of 5.5–10 Å were ideal for the adsorption of PFOA molecules, which aligns with the results for these COFs.^58^ Looking at the other extreme, COF-320 exhibited a decrease in *R*_HLC_ for both functional groups. The pore diameters for COF-320 range between 3.5 Å and 5.5 Å, with the pristine structure showing the largest pores by a small degree. These narrow pores below 5 Å would limit the available positions and orientations for PFOA to successfully be inserted, leading to a decrease in its HLC, and thus lower *R*_HLC_ values.

For COFs where the results for −CF_3_ and −NH_2_ functionalization differ, several can be explained by the pore size distributions. IL-COF-2, BP-COF-1, PPQV-1, and PPQV-2 all see a decrease in *R*_HLC_ with −CF_3_ functionalization, but similar or higher values for −NH_2_ functionalization. CF_3_-functionalized IL-COF-2 and BP-COF-1 both have major peaks in the range of 3–4 Å, while the pristine and NH_2_-functionalized structures have large peaks closer to 5 Å, leading to the decrease in the CF_3_-functionalized structures’ *R*_HLC_ values.

PPQV-1 and PPQV-2 are more difficult to discern. For PPQV-1, we do see a large shift in the largest pore sizes going from the pristine structure to CF_3_-functionalized, with the smallest pores in the functionalized structure falling at 4 Å. However, the CF_3_-functionalized structure still has a large portion of its pores with diameters of 8–10 Å. This likely means that these small 4 Å pores are lowering the average HLC for PFOA, leading to a slightly lower *R*_HLC_ value than anticipated. PPQV-2, on the other hand, shows very similar peaks for all three structures, with pores ranging from 7 to 14 Å, indicating that this is likely not an issue of the pore size excluding the PFOA molecule, but rather an issue of chemistry.

Moving in the other direction, the only COF that shows improvement with −CF_3_ functionalization and not −NH_2_ functionalization is TPTF-COF. Looking at the PSD for TPTF-COF, the pores for the pristine and functionalized structures are all wide enough for PFOA adsorption, with NH_2_-functionalized TPTF-COF having one large peak at approximately 9 Å. This pore size is very similar to that of NH_2_-functionalized COF-300, where we saw an improvement in *R*_HLC_ over the pristine COF. This may indicate that this is an issue of chemistry, rather than the size of the pore.

While the porosity of a pristine COF may give an approximation of its capacity for PFOA adsorption, the change in porosity with functionalization does not relate directly with the resulting *R*_HLC_ values. Instead, we can draw conclusions based on the pore size distributions of the pristine and functionalized COF structures. If functionalization creates excessively small pores (i.e., < 5.5 Å), the PFOA will have limited space to adsorb in the pore, resulting in a low *R*_HLC_ value. With sufficiently large pores, the PFOA will easily be able to adsorb into the pores. Despite this, predicting COF performance by using the pore diameters are not without issues, as we have seen in the cases of CF_3_-functionalized PPQV-2 and NH_2_-functionalized TPTF-COF. In these cases, despite having large pores, PFOA shows significantly lower HLC values, likely due to some fundamental difference in the chemistry of the COFs.

## Discussion

The results found here show promise for the design and prediction of new COF structures for the adsorption of PFOA, one of the most commonly studied PFAS molecules. While the effects of pore size and functionalization have been studied extensively for the adsorption of smaller molecules, such as carbon dioxide, the higher complexity of molecules like PFOA make the interplay between these different properties difficult to predict based on chemical intuition alone. As such, this work lays out the mechanisms by which the pore size and functionalization jointly control the level of PFAS adsorption.

PFOA, as considered here, is only one molecule in a family of several thousand perfluorinated chemicals. Different PFAS molecules can vary in their functional headgroup, such as perfluorocarboxylic acids (PFCAs) and perfluorosulfonic acids (PFSAs), which feature −COOH and −SO_3_H groups respectively. The fluorinated tail can also increase the complexity of a PFAS molecule, with many having increasing tail lengths, as well as branching^75^ and cyclic^76^ structures. Changing any of these structural characteristics of the PFAS molecule will have a profound effect on the adsorptive performance of these COFs, and require further examination of the mechanisms controlling these molecules’ adsorption. Additional computational work may also be necessary to understand the effect of specific binding sites within COF structures as well. We have investigated the effects of binding site and functional positions within the COF-300 structure in our recent work^74^ finding that changes in the electrostatic nature of COF-300 as a result of functionalization can alter the method of PFAS adsorption and the ideal binding site. However, this work did not focus directly on the effects of differing binding sites.

A recent computational study by Johnson et al. focused on the effects of fluorinated tail length in linear PFCAs, as well as nonpolar perfluoroalkanes on their adsorption in the metal organic framework NU-1000.^77^ By varying the chain length for these two classes of PFAS molecules, they found that larger PFCAs tended to adsorb more favorably than short chain PFCAs due to the size and lower hydrophobicity of the smaller molecules. PFCAs and perfluoroalkanes also tended to show different behavior to each other in the presence and absence of water molecules during adsorption.^77^

In future works, it may be pertinent to study the effects of PFAS chain length on the adsorption capability of COFs. The Lennard-Jones parameters for the PFCAs and perfluoroalkanes studied by Johnson et al. have been made available for molecules with chain lengths between 2 and 9 carbon atoms, which will allow researchers to further investigate these molecules.^77^ However, well-defined force field parameters for other classes of PFAS molecules, such as PFSAs and molecules with more complex tail structures, are difficult to find. Future work in this field will require the development of these parameters for new and emerging PFAS molecules.

Similarly, the data analyzed represents only a small number of total possible COF structures. As discussed in our methodology, we chose to study the 120 three-dimensional COFs available in the Curated COF Database. However, other databases of experimentally synthesized COF structures exist, such as the CoRE COF Database.^78^ While there is a significant overlap between their available structures, a large number of COFs are unique to each of the databases. Beyond experimentally synthesized COFs, the ReDD-COFFEE database provides over 250,000 hypothetical COF structures, created through the combination of varied clusters and linkers to form novel frameworks.^79^ The methodology and computational screening approach proposed in this work to investigate this vast number of COFs can help to eliminate structures that are unlikely to perform well for PFAS adsorption, and guide future experimental COF research and synthesis.

It is important to investigate how this computational data compares to existing experimental results. While previously discussed studies looking at the effects of −CF_3_ and −NH_2_ functionalized COFs did not use COF structures from the Curated COF Database^30^ we can compare the trends seen in these functionalized COFs. Ji et al. sought to investigate the effect of different levels of amine functionalization on the adsorption of GenX, an emerging PFAS molecule, from wastewater.^25^ Eight COF structures with varied percentages of functional positions replaced with −NH_2_ groups were synthesized, and the equilibrium GenX removal percentage and adsorption capacity were evaluated in a batch experiment. The results showed that the introduction of amine functionalization improved the GenX removal capability for the COF, however the GenX removal percentage peaked at approximately 20% amine functionalization before depreciating at higher percentages. This aligns with the computational findings discussed here, as higher functional percentages will lead to reduced BET surface areas and average pore diameters, limiting the PFAS adsorption capability.

An experimental study by Sun et al. focused on the introduction of trifluoromethyl functional groups to a nitrogen-based COF for the solid-phase microextraction of various PFAS molecules from milk samples.^28^ The CF_3_-functionalized COF served as a coating for fibers being applied in microextraction techniques. These samples were characterized with ultrahigh performance liquid chromatography, and tested for repeated extraction. The addition of the trifluoromethyl functional group resulted in high extraction capability for a wide range of PFAS molecules, with the CF_3_–COF coated fibers able to be used repeatedly with satisfactory results.^28^ Similarly, this is in agreement with the findings outlined in this study.

Finally, Song et al. synthesized a multifunctionalized COF structure, utilizing −NH, −CO, and −C_4_F_9_ functional groups to take advantage of electrostatic and fluorophilic interactions with PFAS molecules.^29^ Fourteen molecules, including anionic, cationic, and zwitterionic PFAS molecules, were used to study the ability of this multifunctionalized COF to adsorb different types of PFAS, both in microextraction conditions and in real water samples. This combination of functionals was found to adsorb various PFAS molecules more effectively than the three functional groups alone, as well as the combination of −NH and −CO functional groups.^29^ This aligns with the previously discussed experimental studies and our computational results, but further, this research can serve as an inspiration for future computational works on mixed-functional COFs for PFAS adsorption.

## Conclusions

With growing concerns over the accumulation of fluorinated “forever chemicals” in the environment, novel porous materials for the adsorption of these chemicals are in high demand. In this work, we used molecular simulations to investigate the adsorption of perfluorooctanoic acid (PFOA) in 96 three-dimensional COF structures. We also functionalized 10 of these structures with trifluoromethyl (−CF_3_) and amine (−NH_2_) groups to study their effects on adsorption. Of the 96 COFs studied, we found that the vast majority exhibited hydrophobic behavior based on the Henry’s law coefficient of water in the COF pores being in the range of 10^–3^–10^–6^ mol kg^–1^ Pa^–1^. By measuring the ratio between the Henry’s law coefficients of PFOA and water (*R*_HLC_), we showed that a large number of COFs, particular nitrogen-based structures, have high potential for PFOA adsorption. In our functionalized COFs, we saw that both the −CF_3_ and −NH_2_ functional groups have the ability to improve the *R*_HLC_ of a COF, but it is heavily dependent on the pore sizes before and after functionalization.

Further tests could be performed on COFs with larger pores that exhibited low *R*_HLC_ values in the pristine case, to see if functionalization with −CF_3_ and −NH_2_ would significantly improve the adsorption of PFOA. COFs with larger pore sizes may also allow for the study of multifunctionalized COFs, to combine the benefits of several functional groups. Investigating different PFAS molecules with novel functional head groups and fluorinated tail structures may also elucidate the changes in the mechanisms dominating adsorption in variously functionalized COFs.

## Supplementary Material







## Notes

Porosity, Henry’s law coefficients (PFOA and water); *R*_HLC_ values for 96 pristine COFs; 10 COFs functionalized with trifluoromethyl and amine groups (XLSX)
Example files for COF-300; calculation of Henry’s law coefficients; geometric and electronic optimization of functionalized structures; pore size analysis (ZIP)
Porosities for 10 selected COFs selected calculated using PoreBlazer v4.0, RASPA, Zeo++ respectively (PDF)
