# Supplementary material for: Design Principles for PFAS Adsorption in Three-Dimensional Covalent Organic Frameworks
Source: J Phys Chem C Nanomater Interfaces. 2026 Apr 8;130(15):5721–32. doi: 10.1021/acs.jpcc.6c01608 (PMC13093484; doi:10.1021/acs.jpcc.6c01608)
Supplement: Supplementary file 3 [file jp6c01608_si_003.pdf]

Supporting Information: Design Principles for PFAS Adsorption in Three-Dimensional Covalent Organic Frameworks

Daniel D. Mottern,[†] Andrei L. Kolesnikov,[†,‡] Gennady Y. Gor,[*,†] and Joshua Young[†,¶]

†*Otto H. York Department of Chemical and Materials Engineering, New Jersey Institute of Technology, Newark, New Jersey 07102, United States*

‡*Institut für Nichtklassische Chemie e. V., Permoserstraße 15, 04318 Leipzig, Germany*

¶*Matlantis Inc., 1 Broadway, Cambridge, MA, 02142, United States*

E-mail: gor@njit.edu

Table S1: Calculated porosities for ten COFs selected for functionalization. Calculations were performed using the PoreBlazer v4.0, RASPA, and Zeo++ softwares respectively.

**COFS**	**PoreBlazer v4.0**	**RASPA**	**Zeo++**
**COF-300**	0.58	0.565	0.572
**COF-320**	0.21	0.223	0.196
**BF-COF-1**	0.73	0.689	0.711
**BP-COF-1**	0.60	0.591	0.619
**BP-COF-4**	0.74	0.716	0.728
**IL-COF-2**	0.32	0.340	0.347
**IL-COF-3**	0.37	0.351	0.385
**PPQV-1**	0.59	0.597	0.604
**PPQV-2**	0.60	0.623	0.617
**TPTF-COF**	0.63	0.668	0.646
